# Traditional Chinese Medicine Preparation Combined Therapy May Improve Chemotherapy Efficacy: A Systematic Review and Meta-Analysis

**DOI:** 10.1155/2019/5015824

**Published:** 2019-06-20

**Authors:** Jiaming Wu, Yun Liu, Cantu Fang, Lixian Zhao, Lizhu Lin, Liming Lu

**Affiliations:** ^1^Zhongshan Affiliated Hospital, Guangzhou University of Chinese Medicine (Zhongshan Hospital of Traditional Chinese Medicine), Zhongshan 528400, China; ^2^South China Research Center for Acupuncture and Moxibustion, Medical College of Acu-Moxi and Rehabilitation, Guangzhou University of Chinese Medicine, Guangzhou 510006, China; ^3^Guangzhou University of Chinese Medicine, Guangzhou 510000, China; ^4^The First Affiliated Hospital of Guangzhou University of Chinese Medicine, Guangzhou 510000, China; ^5^Clinical Research Center, South China Research Center for Acupuncture and Moxibustion, Medical College of Acu-Moxi and Rehabilitation, Guangzhou University of Chinese Medicine, Guangzhou 510000, China

## Abstract

**Background:**

Whether traditional Chinese medicine preparation combined therapy can improve the efficacy of chemotherapy is controversial. This meta-analysis evaluates the efficacy of traditional Chinese medicine preparation combined with chemotherapy.

**Method:**

Three databases were searched from inception through August 2018. Eligible randomized controlled trials (RCTs) involving the combined treatment of chemotherapy and traditional Chinese medicine preparation compared to chemotherapy alone for treating cancer were retrieved. The methodological quality of the included RCTs was assessed with Cochrane Collaboration's risk of bias assessment tool. Meta-analysis was adopted to make comprehensive comparisons between the experimental and control groups.

**Results:**

Four RCTs were included in this review, comprising 256 subjects. The majority of the RCTs were judged as being of poor methodological quality. Meta-analysis showed that the combination of traditional Chinese medicine preparation and chemotherapy appeared to be more effective than chemotherapy alone, for the treatment of cancer, as assessed by the disease control rate (RR: 1.41, 95% CI: 1.11 to 1.79) and the objective response rate (RR: 2.71, 95% CI: 1.28 to 5.77). There were no statistically significant differences between the groups in terms of bone marrow suppression (RR: 0.88, 95% CI: 0.57 to 1.37) or gastrointestinal reaction (RR: 1.12, 95% CI 0.75 to 1.69).

**Conclusions:**

Traditional Chinese medicine preparation combined with chemotherapy may improve objective response rates and disease control rates more than chemotherapy alone. The evidence that combined traditional Chinese medicine preparation can reduce the side effects of chemotherapy is insufficient. More rigorous randomized controlled trials are needed to confirm these conclusions.

## 1. Introduction

Cancer is a serious threat to human health and life. Data from the latest global cancer statistics show that there will be 18.1 million new cancer cases and 9.6 million cancer deaths in 2018 [1]. Radiotherapy and chemotherapy are the main treatments for cancer. However, chemotherapy's efficacy has reached a bottleneck, and it may also cause bone marrow suppression, gastrointestinal reactions, and other side effects [2, 3].

In China, many cancer patients are treated with Chinese medicine such as Chinese medicine preparation, acupuncture, cupping, Taichi, and massage. Among them, the curative effect of Chinese medicine preparation (e.g., herbal medicine and patent medicine) combined with chemotherapy is remarkable. Many studies have found that the combination of chemotherapy and traditional Chinese medicine preparation improves chemo sensitivity and mitigates the side effects of chemotherapy. A phase II trial of the botanical formulation PHY906 found that patients in the combined Chinese medicine group had higher disease control rates and median progression-free survival times [8]. A study of the traditional Chinese medicine rikkunshito combined with chemotherapy found that the traditional Chinese medicine preparation combined group had a higher one-year survival rate [4]. Many clinical studies have demonstrated that traditional Chinese medicine preparation can reduce the incidence of bone marrow suppression and gastrointestinal reactions in chemotherapy [5, 9].

Based on the above findings, researchers have conducted systematic reviews of Chinese medicine preparation in the treatment of cancer. The first systematic review of this field was published in 2013; it evaluated 13 randomized controlled trials and found that Chinese medicine preparation can improve tumor response rate, one-year survival, and quality of life in cancer patients [10]. However, most of the studies in this review used small samples and were of low quality. This may have led to erroneous conclusions. A systematic review of 1,843 patients found that combined treatment with traditional Chinese medicine preparation significantly reduced chemotherapy-related vomiting. However, no other indicators of tumor efficacy were reported [11]. The third systematic review obtained different results; it indicated that Chinese medicine injections combined with chemotherapy does not achieve better clinical effects, nor does it reduce nausea and vomiting [12].

This meta-analysis systematically updates new findings in this field on the basis of previous research results. We address the following questions:

(1) Can combination with traditional Chinese medicine preparation increase the sensitivity of chemotherapy?

(2) Can combination traditional Chinese medicine preparation reduce the side effects of chemotherapy?

## 2. Methods

### 2.1. Search Strategy

A systematic search was conducted to identify published RCTs on CHM treating patients with cancer via the following electronic databases, from inception to August 2018: MEDLINE, EMBASE, and The Cochrane Central Register of Controlled Trials. The search strategy is provided in Appendix I.

### 2.2. Selection Criteria

Studies meeting the following criteria were included:

(1) They claimed RCTs with baseline data without significant differences in clinical characteristics, among both the experimental and the control groups.
(2) The subjects of both groups were patients diagnosed with cancer.
(3) The experimental group received CHM combined with other active treatments, which was the same as was given to the control group.
(4) Studies investigated at least one of the outcomes listed below:
  (I) Clinical benefit, number of patients with complete response (CR), partial response (PR), stable disease (SD), or progressive disease (PD) evaluated with the WHO scale.
  (II) Conventional therapy-induced toxicity events, including anorexia, nausea, vomiting, bone marrow suppression, and changes in haemoglobin, platelets, and white blood cells (WBCs).

### 2.3. Risk of Bias Assessment

Risk bias analysis of the studies was performed independently by W.J.M and L.Y. using the Cochrane Risk of Bias Assessment Tool for the following criteria: random sequence generation, allocation concealment, blinding of outcome assessment, incomplete outcome data, selective reporting, and other bias. In cases when the reviewers' opinions varied, a consensus was reached through discussion. Evidence from research studies was ranked as having either “high,” “low,” or “unclear” risk of bias.

### 2.4. Data Synthesis

All statistical analyses were performed with Reviewer Manager 5.3 (Cochrane Collaboration, Oxford, UK) to quantify and compare the efficacy outcomes of the experimental versus the control groups. The impact of CHM on dichotomous data was expressed as a risk ratio (RR); a random-effects model was employed in cases in which the study of heterogeneity (I^2^) was larger than 50%. Sensitivity analyses were conducted for primary outcomes.

## 3. Results

### 3.1. Search Results

**Figure 1:**
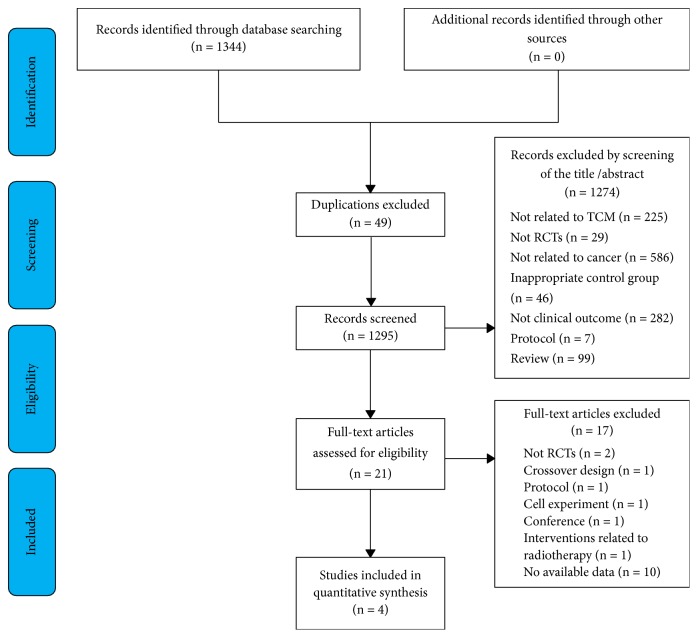
Flowchart of the trial selection process.

Figure 1 depicts the study's inclusion and exclusion process. We identified potentially eligible articles through the relevant databases. First, forty-nine duplicates were removed, and 1,274 papers were excluded after screening their titles and abstracts. After screening the full texts of the included articles, 17 studies were excluded for the following reasons: crossover design (n = 1), protocol (n = 1), cell experiment (n = 1), not RCTs (n = 2), conference papers (n =1), interventions related to radiotherapy (n=1), or no available data (n = 10). Finally, 4 studies [1–4, 8] were included for further analysis (Figure 1).

### 3.2. Description of Studies

The characteristics of the included studies in this review are shown in Table 1. Of the four studies, two were published in Chinese and two in English; all of the studies were conducted in China. Together, these studies involved a total of 256 subjects, with 129 being in the treatment arm and 127 in the control arm. All subjects included in this study had been diagnosed with cancer. All included trials used a two-armed, parallel group design. Subjects in all studies had received CHM combined with chemotherapy in the treatment arm and chemotherapy alone in the control arm [4–7]. Of the included studies, three reported disease control rates [4–6], two reported objective response rates [5, 6], four reported bone marrow suppression, including anaemia, neutropenia, thrombocytopenia, or leukopenia [4–7], and two studies reported gastrointestinal reactions including anorexia, nausea, or vomiting [4, 5].

### 3.3. Quality of the Included Studies

**Figure 2:**
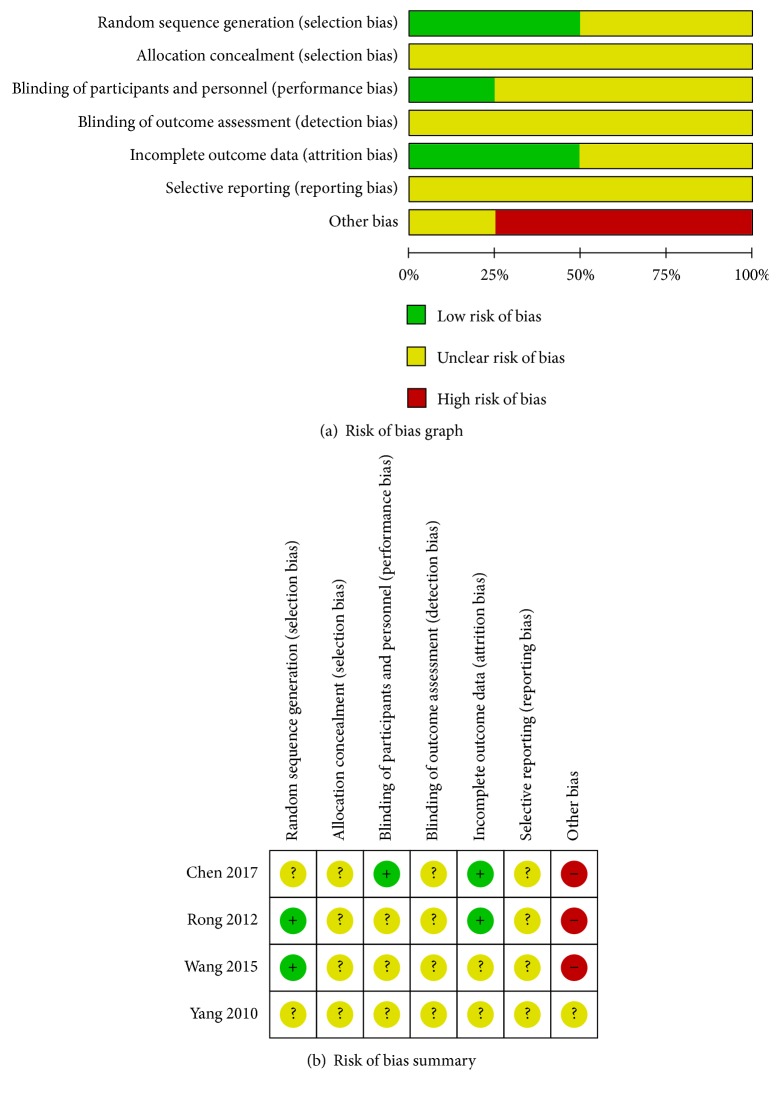
Assessment of risk of bias: (a) risk of bias graph and (b) risk of bias summary.

The ROB results for each included RCT are presented in Figure 2. With regard to random sequence generation and incomplete outcome data, two studies were rated low-risk, and another two were rated unclear risk. With regard to the binding of participants and personnel, only one study was rated low-risk, and the remaining were rated unclear risk. All included studies were rated unclear risk for allocation concealment, blinding of outcome assessment, and selective reporting. Regarding other biases, only one study was rated unclear risk, and the remainder were rated high-risk.

### 3.4. Disease Control Rate

**Figure 3:**
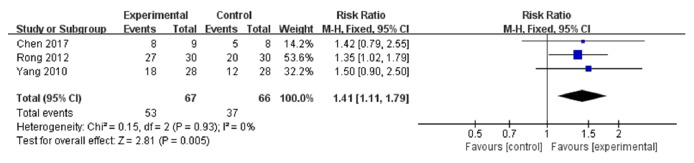
Forest plot of disease control rate.

The primary outcome measure, disease control rate, is shown in Figure 3. Three trials [4–6] (133 participants, 67 in the experimental group, and 66 in the control) were used in this analysis. The pooled results indicated that the experimental group had better effects than the control group (RR: 1.41, 95% CI: 1.11 to 1.79), with no heterogeneity among the studies (I^2^ = 0%, *P*=0.93).

### 3.5. Objective Response Rate

**Figure 4:**
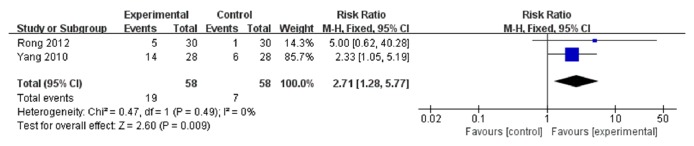
Forest plot of objective response rate.

The results of the meta-analysis of the secondary outcome measure and objective response rate are shown in Figure 4. Two trials [5, 6] (116 participants, 58 in the experimental group, and 58 in the control) were adopted in this group. The pooled results indicated that the experimental group had better effects than the control group (RR: 2.71, 95% CI: 1.28 to 5.77), with no heterogeneity among the studies (I^2^ = 0%,* P*=0.49).

### 3.6. Bone Marrow Suppression

**Figure 5:**
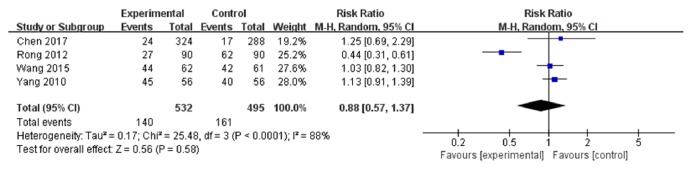
Forest plot of bone marrow suppression.

A total of 4 trials [4–7] including 1,027 person-times (532 in the experimental group and 495 in the control) were involved in this group (Figure 5). A random-effects model was used to calculate the combined RR (0.88) and 95% CI (0.57 to 1.37) due to the heterogeneity between studies (I^2^=88%,* P*<0.0001).

### 3.7. Gastrointestinal Reaction

**Figure 6:**
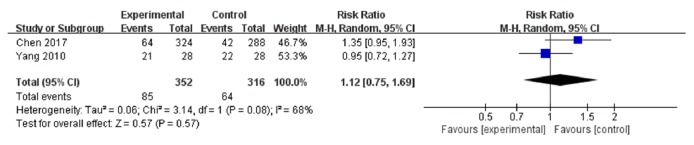
Forest plot of gastrointestinal reaction.

Two trials [4, 5] including 668 person-times (352 in the experimental group and 316 in the control) were involved in this group (Figure 6). A random-effects model was used to calculate the combined RR (1.12) and 95% CI (0.75 to 1.69) due to the heterogeneity between studies (I^2^=68%,* P*=0.08).

### 3.8. Sensitivity Analysis

**Figure 7:**
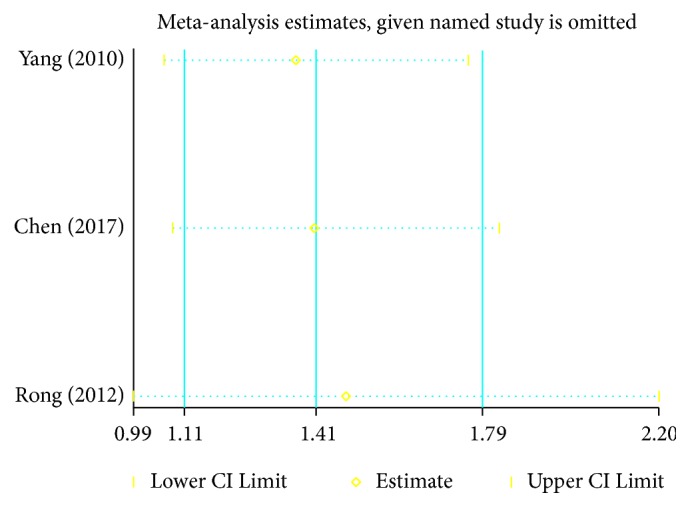
Sensitivity analysis.

Sensitivity analysis was performed on the trials. It reported disease control rates and yielded a similar result, with no heterogeneity (Figure 7).

## 4. Discussion

This meta-analysis included 4 RCTs comparing chemotherapy alone versus chemotherapy combined with traditional Chinese medicine preparations; 316 participants were included. The result of the meta-analyses suggested that TCM preparations had a beneficial effect on chemotherapy efficacy. The disease control and objective response rates of the combined group were significantly higher than those of the control group. Furthermore, there was no between-trial heterogeneity in the meta-analyses of the primary outcomes. However, there was no obvious advantage in the combined group for the incidence of bone marrow suppression and gastrointestinal reactions, and there was heterogeneity between the groups. Sensitivity analysis by removing high-risk biases reached similar results, reflecting the reliability and robustness of our results.

For the quality of the included studies, 60% were rated as low-risk in terms of random sequence generation. Only 20% of the included studies were rated as low-risk in the binding of participants and personnel. With regard to incomplete outcome data, 60% of the research was considered to be low-risk. Because of the lack of detailed information, all of the included studies were rated as having unclear risk of allocation concealment, blinding of outcome assessment, and selective reporting. 4 studies were rated as high-risk because the drug manufacturer had been mentioned in the article. The bias of appeal may have affected the credibility of the results. We acknowledge the limitations of these results and hope for more high-quality research in the future.

### 4.1. Recommendations for Practice

Recent studies have found that it is difficult to improve the efficacy of cancer chemotherapy, and increasing the dosage only yields additional side effects. The results of our study suggest that the combination of traditional Chinese medicine preparations and chemotherapy may improve the efficacy of chemotherapy, without increasing adverse reactions. For clinical implications, Chinese medicine preparations can be used in combination with chemotherapy, improving its curative effect. The limited evidence cannot confirm that Chinese medicine preparations reduce bone marrow suppression or gastrointestinal reactions due to chemotherapy.

### 4.2. Research on Mechanisms

P-glycoprotein (P-gp) is a drug transporter on the cell membrane. Previous studies have shown that P-glycoprotein (P-gp) induces chemotherapeutic drug resistance by reducing intracellular drug concentration [13–15]. Chinese medicine preparations may be particularly effective in chemotherapeutic resistance mediated by elevated expression of P-glycoprotein (P-gp). Several researchers have used traditional Chinese medicine as a P-gp reversal agent, both* in vitro* and in rat MDR tumor models. Gui et al. [16] reported that the apoptotic degree of K562/AO2 cells increased from 4.81% to 15.31% in the presence of matrine. RT-PCR analysis showed that P-gp protein level was downregulated, suggesting that matrine might reverse MDR (and enhance chemical sensitivity) by downregulating P-gp levels. In another study [17], Rh2 ginsenoside was added to MDR breast cancer MCF7/ADM cells, and then the cell resistance to both doxorubicin (DOX) and 5-fluorouracil (5fu) was tested. It was found that Rh2 ginsenoside can reduce P-gp activity and reverse the MDR of cancer cells. In an animal experiment [18], mice with tumors were divided into 4 groups and treated with either saline, vincristine (VCR) alone, curcumin alone, or VCR and curcumin. The tumor tissue weight and P-gp protein levels were measured after 2 weeks. The results showed that the tumor weight and the P-gp protein levels in the VCR/curcumin combination group were significantly lower than those in the other groups.

O6-methylguanine-DNA methyltransferase (MGMT) is a DNA alkylation damage repair enzyme. It can eliminate DNA damage caused by alkylating agents and can also enhance cancer cells' tolerance to chemotherapy drugs. Many studies have confirmed that overexpression of the O6-methylguanine-DNA methyltransferase (MGMT) gene is an important mechanism of cancer resistance to chemotherapeutic drugs [19–21]. A clinical study published by Rong Z [6] found that the methylation rate of MGMT genes in the plasma of cancer patients treated with dujieqing oral liquid is lower than those treated with chemotherapy alone. This suggests that traditional Chinese medicine may improve chemosensitivity by regulating MGMT activity. This may be another mechanism of traditional Chinese medicine preparation which could improve the efficacy of chemotherapy.

### 4.3. Limitations

Our study has several limitations. First, the sample size of our meta-analysis was too small, and this may have affected the accuracy of the results. We conducted sensitivity analysis to confirm that the results are stable and reliable. Second, inconsistent treatment with Chinese medicine may have led to heterogeneity in the meta-analysis.

### 4.4. Conclusion

TCM preparations combined with chemotherapy may improve objective response rates and disease control rates, compared to chemotherapy alone. The evidence that combined Chinese medicine preparations can reduce the side effects of chemotherapy is insufficient. More rigorous randomized controlled trials are needed to confirm these findings.

## Figures and Tables

**Table 1:** Characteristics of included studies.

Study ID	Participants	Sample	Intervention		Outcomes
		(Experimental/Control)	Experimental	Control	
Chen 2017 [4]	non-small-cell lung cancer	9/8	cisplatin + gemcitabine + rikkunshito	cisplatin + gemcitabine	①③④
Yang 2010 [5]	non-Hodgkin's lymphoma	28/28	Chemotherapy + ligustrazine	chemotherapy alone	①②③④
Rong 2012 [6]	middle and late stage tumor	30/30	chemotherapy + Dujieqing Oral Liquid	chemotherapy alone	①②③
Wang 2015 [7]	lung cancer	62/61	Chemotherapy + Yiqi Jianpi Recipe	chemotherapy alone	③

①: disease control rate; ②: objective response rate; ③: bone marrow suppression; ④: gastrointestinal reactions.

## Data Availability

The data used to support the findings of this study are available from the corresponding author upon request.
